# Ultrasound for the detection of the pyramidal lobe of the thyroid gland

**DOI:** 10.1007/s40618-022-01748-z

**Published:** 2022-02-14

**Authors:** A. Freilinger, K. Kaserer, G. Zettinig, P. Pruidze, L. F. Reissig, T. Rossmann, W. J. Weninger, S. Meng

**Affiliations:** 1 Division of Anatomy, Center for Anatomy and Cell Biology, Medical University Vienna, Waehringer Str. 13, 1090 Vienna, Austria; 2 Laboratory Kaserer, Koperek und Beer OG, Reisnerstraße 5, 1030 Vienna, Austria; 3 Thyroid Center “Schilddrüsenpraxis Josefstadt”, Laudongasse 12, 1080 Vienna, Austria; 4 Department of Neurosurgery, Neuromed Campus, Kepler University Hospital, Wagner-Jauregg-Weg 15, 4020 Linz, Austria; 5 Department of Radiology, Hanusch Hospital Vienna, Heinrich-Collin-Straße 30, 1140 Vienna, Austria

**Keywords:** Pyramidal lobe, Ultrasound, Thyroid imaging, Thyroid surgery

## Abstract

**Purpose:**

The pyramidal lobe (PL) is an ancillary lobe of the thyroid gland that can be affected by the same pathologies as the rest of the gland. We aimed to assess the diagnostic performance of high-resolution sonography in the detection of the PL with verification by dissection and histological examination.

**Methods:**

In a prospective, cross-sectional mono-center study, 50 fresh, non-embalmed cadavers were included. Blinded ultrasound examination was performed to detect the PL by two investigators of different experience levels. If the PL was detected with ultrasound, dissection was performed to expose the PL and obtain a tissue sample. When no PL was detected with ultrasound, a tissue block of the anterior cervical region was excised. An endocrine pathologist microscopically examined all tissue samples and tissue blocks for the presence of thyroid parenchyma.

**Results:**

The prevalence of the PL was 80% [40/50; 95% CI (68.9%; 91.1%)]. Diagnostic performance for both examiners was: sensitivity (85.0%; 42.5%), specificity (50.0%; 60.0%), positive predictive value (87.2%; 81.0%), negative predictive value (45.5%; 21.0%) and accuracy (78.0%; 46.0%). Regression analysis demonstrated that neither thyroid parenchyma echogenicity, thyroid gland volume, age nor body size proved to be covariates in the accurate detection of a PL (*p* > .05).

**Conclusion:**

We report that high-resolution ultrasound is an adequate examination modality to detect the PL. Our findings indicate a higher prevalence than previously reported. Therefore, the PL may be regarded as a regular part of the thyroid gland. We also advocate a dedicated assessment of the PL in routine thyroid ultrasound.

## Background

The pyramidal lobe (PL) is an accessory lobe of the thyroid gland, consisting of thyroid tissue that remains at the caudal end of the obliterated thyroglossal duct. Its varying prevalence in previous studies ranged from 35.7 to 60.0% in cadaver studies [1–5] and from 12.3 to 61.0% in surgical studies [6–10], as showcased in Table 1.

**Table 1:** Prevalence of the pyramidal lobe in previous cadaver and surgical studies

Study	Pyramidal lobe
Post-mortem-based studies	
Braun et al. [1]	55.2% (32/58)
Ranade et al. [2]	58.1% (61/105)
Ozgur et al. [3]	60.0% (24/40)
Prakash et al. [4]	35.7% (25/70)
Milojevic et al. [5]	55.2% (32/58)
Surgery-based studies	
Geraci et al. [6]	12.3% (74/604)
Zivic et al. [7]	61.0% (61/100)
Kim et al. [8]	59.8% (79/132)
Ryu et al. [9]	60.0% (81/135)
Irawati et al. [10]	36.9% (38/103)

There is no gender difference in prevalence, length, width, or thickness of the PL [5].

A fibrous or muscular band descending from the hyoid bone to the tip of the PL, also referred to as levator glandulae thyroideae muscle was described in 17.5–49.5%. It is believed to be a remainder of the thyroglossal duct and is of yet unknown clinical significance [2, 3, 5].

On the contrary, the PL can be affected by the same pathologies as the rest of the thyroid gland. Hence, it may be afflicted in generalized thyroid disease as well as multifocal thyroid carcinoma [6, 7, 10]. The PL has been reported as the primary site of papillary and folliculary thyroid carcinoma in multiple case series [11–14]. Although rare, papillary thyroid carcinoma arising from the PL was significantly associated with a more frequent lymphatic invasion, a higher tumor stage and a more advanced American Joint Committee on Cancer (AJCC) stage when compared to papillary carcinoma found elsewhere in the thyroid gland. Prevalence of papillary thyroid cancer stemming from or afflicting the PL was 4.4% (49/1107) [13]. Owing to its mostly delicate size, the PL may be overlooked during surgery and may be a potential site for recurrent disease [15–17]. Sencar et al*.* found that 181(10.4%) of 1740 patients with differentiated thyroid cancer treated with total thyroidectomy showed pyramidal lobe residue on postoperative thyroid scintigraphy. These patients showed significantly higher levels of thyroglobulin and significantly lower levels of thyroid stimulating hormone (TSH) on follow-up [18]. While the secretion of thyroglobulin can complicate postoperative follow-up, remnant thyroid tissue may interfere with radioiodine treatment as it competes with tumor cells for the uptake of radioactive ^131^I [19, 20].

Therefore, reliable and accessible routine imaging of the PL is of interest. Radiologic prevalence of the PL was reported ranging from 21.0 to 58.5% using ultrasound and 44.6–56.3% using computed tomography (CT) respectively [8, 9, 21–24]. Prevalence of the PL on thyroid scintigraphy was reported as being 13–41% [1, 25]. However, scintigraphy does not seem to be suitable in visualizing a PL, as it also strongly depends on underlying pathologies of the thyroid gland [1].

Ultrasound examination of the anterior cervical region is part of basic evaluation of both benign and malignant thyroid disease [26, 27]. When compared to surgical prevalence, it was shown that ultrasound has a sensitivity of 81.0–85.2% and a specificity of 79.2–81.5% in prior studies, comparing unfavorably with CT (sensitivity 91.4% and specificity 94.4%). However, CT is not routinely performed in every patient presenting with thyroid pathology and associated with radiation exposure and the application of iodine based contrast medium [8, 9].

We hypothesized that the PL can be reliably detected and assessed in routine ultrasound examination with high-resolution ultrasound systems and aimed to establish diagnostic values verified by anatomical and histological confirmation. Furthermore, we investigated whether a novice ultrasound examiner was able to detect the PL.

## Materials and methods

This study was carried out as a prospective, cross-sectional mono-center study. Approval of the institutions ethics committee was obtained (ECS Nr. 1921/2019).

Fifty randomly recruited, fresh, non-frozen, non-embalmed cadavers were included. All persons had declared to donate their bodies for scientific and academic purposes prior to death. Body donations with documented thyroid surgery, scars in the anterior cervical region, or other evidence of trauma or surgical intervention to this region were not included. Mean age was 81.3 ± 10.2 years and all individuals lived in the previously iodine deficient area of Austria where iodine supplementation was introduced in 1963 [28].

Two investigators with differing amounts of experience performed blinded ultrasound of the anterior cervical region. Examiner I is a radiologist specialized in ultrasound examination. Examiner II acquired basic ultrasound skills during medical school and student courses. A clinical ultrasound system (Aplio i800, Canon Medical Systems Zoetermeer, Netherlands) with an 18 MHz, high-resolution, linear transducer (i18LX5) was used. The anterior neck region cranial of the thyroid gland was examined primarily in an axial scanning plane. Relevant ultrasound parameters of the thyroid gland and of the PL (width, depth, site of origin, distance to the midline) were assessed by examiner I. Thyroid lobe volume was calculated using the formula height × width × depth × π/6 according to Brunn [29].

If detected by examiner I, the anterior neck region was dissected to expose the PL. Parameters of the PL (length, width, distance to midline) were measured in situ and a tissue sample was obtained. Distance to midline was always measured at the point closest to midline.

In the case of no visualization of the PL on ultrasound by examiner I, a tissue block of the anterior cervical region was harvested. This was also performed if the PL was identified with ultrasound but not detected upon consequent dissection. The borders of the tissue block were:

- Caudal: Thyroid isthmus.
- Cranial: Approximately 1 cm cranial of the thyroid isthmus.
- Lateral: lateral border of the infrahyoidal muscles.

Each block contained the tissue layers from the superficial fascia to the thyroid cartilage, ensuring the microscopic detection of a thyroid tissue missed with ultrasound.

All tissue samples were transferred to a pathology laboratory where they were prepared for routine pathological analysis. These samples did not include tissue from the thyroid lobes or the isthmus. Standard hematoxylin and eosin staining was applied. A pathologist examined the slides to evaluate the presence of thyroid tissue.

Statistical analysis was carried out with IBM SPSS^®^ 22.0 Statistical Software System (SPSS Inc. Chicago, IL). The level of significance was set at *α* = 5% so that results in hypothesis testing with *p* ≤ 0.05 were considered significant. We employed descriptive statistics (M ± SD, Md, IQR, confidence intervals, absolute frequencies and proportions) to report relevant parameters. The Kolmogorov–Smirnov test was performed to assess the normal distribution of continuous, metric variables. To examine the difference of mean ranks of non-normally distributed parameters we used a two-tailed Wilcoxon signed-rank test. Binary logistic regression analysis was carried out to investigate potential covariates in the accurate detection of the PL using ultrasound.

## Results

The PL was present in 80% (40/50) as confirmed by histopathological investigation. In 4% (2/50), the PL showed separation from the isthmus and was located ventrally on the thyrohyoid membrane. The levator glandulae thyroideae muscle was observed in 14% (7/50) including two cases (2/50, 4%) associated with a thyroglossal duct cyst. Neither thyroid nodules nor cancer were detected in a pyramidal lobe.

Histopathological examination showed thyroid tissue in 97.5% (39/40). In the remaining case, a PL was detected on ultrasound and consequent dissection, while the histopathological workup revealed longitudinally arranged muscular, fibrous, and fatty tissue, which corresponded to levator glandulae thyroideae muscle, hence being the cranial extension of the PL. Specimen and PL characteristics are presented in Table 2.

**Table 2:** Characteristics of the study population and the pyramidal lobe

Parameter	*n* = 50
Sex	
Female	28 (56%)
Male	22 (44%)
Age (years)	81.3 ± 10.2
Body height (cm)	165.6 ± 10.7
PL present	40 (80%), 95% CI (68.9%; 91.1%)
PL only	31 (62%)
PL separated from thyroid isthmus	2 (4%)
PL with levator glandulae thyroideae muscle only	5 (10%)
PL with levator glandulae thyroideae muscle associated with thyroglossal duct cyst	2 (4%)
PL absent	10 (20%)

Data are presented in values (M ± SD), frequencies and proportions (%)

### Diagnostic indices

The PL was reported in 78% (39/50) by examiner I. Detection rates for examiner I were 34 true-positives, 5 false-positives, 5 true-negatives and 6 false-negatives, respectively.

Examiner II observed the PL in 42% (21/50). Detection rates for examiner II were 17 true-positives, 4 false-positives, 6 true-negatives and 23 false-negatives, respectively. Diagnostic indices are listed in Table 3.

**Table 3:** Diagnostic indices for the sonographic detection of the PL

	Examiner I	Examiner II
Sensitivity	85.0% (34/40)	42.5% (17/40)
Specificity	50.0% (5/10)	60.0% (6/10)
PPV	87.2% (34/39)	81.0% (17/21)
NPV	45.5% (5/11)	20.7% (6/29)
Accuracy	78.0% (39/50)	46.0% (23/50)

*PPV*: positive predictive value,* NPV*: negative predictive value

To assess whether any parameters interfere with the diagnostic accuracy, binary logistic regression was conducted. Neither thyroid parenchyma echogenicity, nor thyroid gland volume, nor age, nor body size proved to be covariates in the accurate detection of a PL, as defined by the true-positive and true-negative predictions by examiner I (*p* > 0.05, Nagelkerke’s *R*^2^ = 9.6%). The results of the regression analysis are presented in Table 4.

**Table 4:** Results of binary logistic regression analysis

Variable	*p*	OR	95% CI OR	
			LL	UL
Thyroid gland volume	0.739	1.011	0.949	1.076
Thyroid gland parenchyma echogenicity	0.637			
Mild hypoechogenicity	0.445	2.154	0.301	15.424
Marked hypoechogenicity	0.557	0.512	0.055	4.797
Age-related degenerative changes	0.598	1.608	0.275	9.399
Age	0.233	0.952	0.878	1.032
Height	0.269	0.957	0.884	1.035

*OR*: odds ratio,* CI*: confidence interval,* LL*: lower limit,* UL*: upper limit

### PL characteristics on ultrasound

The location of the 34 true-positive cases identified with ultrasound by examiner I was on the left side in 16 cases, midline in 4 cases and on the right side in 14 cases, respectively. Mean PL width, depth and distance to midline were 6.92 ± 3.18 mm, 2.06 ± 0.91 mm and 5.31 ± 4.17 mm respectively. Median volume of the thyroid gland was 15.97 (IQR12.39–19.48) cm^3^ in females and 22.76 (IQR 15.24–33.88) cm^3^ in males. The characteristics of the thyroid gland parenchyma assessed with ultrasound are presented in Table 5.

**Table 5:** Ultrasound-based thyroid gland characteristics and focal thyroid gland lesions

TG characteristics	*n* = 50
Volume (cm^3^)	
Female	15.97 (12.39–19.48)
Male	22.76 (15.24–33.88)
Size	
Normal	35 (70%)
Enlarged (*f* > 18 cm^3^, *m* > 25 cm^3^)	15 (30%)
Echogenicity	
Normal	14 (28%)
Mild hypoechogenicity	13 (26%)
Marked hypoechogenicity	6 (12%)
Age-related degenerative changes	17 (34%)
Focal lesions	
No focal lesion	19 (38%)
Singular	5 (10%)
Multiple	26 (52%)
Focal lesion morphology	
No focal lesion	19 (38%)
Nodular	21 (42%)
Cystic/nodular	5 (10%)
Cystic	2 (4%)
Macrocalcification	3 (6%)

Data are presented in Values (Md, IQR), Frequencies and proportions (%)

### PL characteristics after dissection

Dissection was performed in 39 cases. The PL was exposed in 33 cases, including the two PL separated from the thyroid isthmus. Location was on the left side in 15 cases, midline in 7 cases and the right side in 11 cases, respectively. In the remaining six cases, the PL could not be exposed on dissection and the tissue block was extracted accordingly after realignment of the infrahyoid muscles. The PL was detected in one tissue block on histopathological examination. Mean PL length, width and distance to midline measured in situ were 36.95 ± 13.26 mm, 6.93 ± 3.11 mm and 3.04 ± 3.21 mm, respectively.

### Ultrasound vs. macroscopic anatomy

**Fig. 1:**
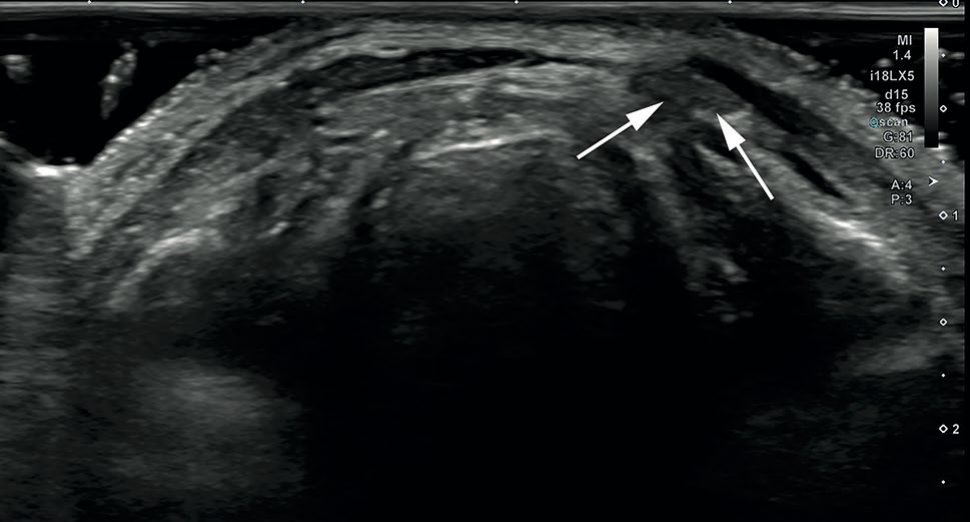
Transverse gray-scale sonogram of an 80 year old male cadaver showing a left-sided PL (arrows)

**Fig. 2:**
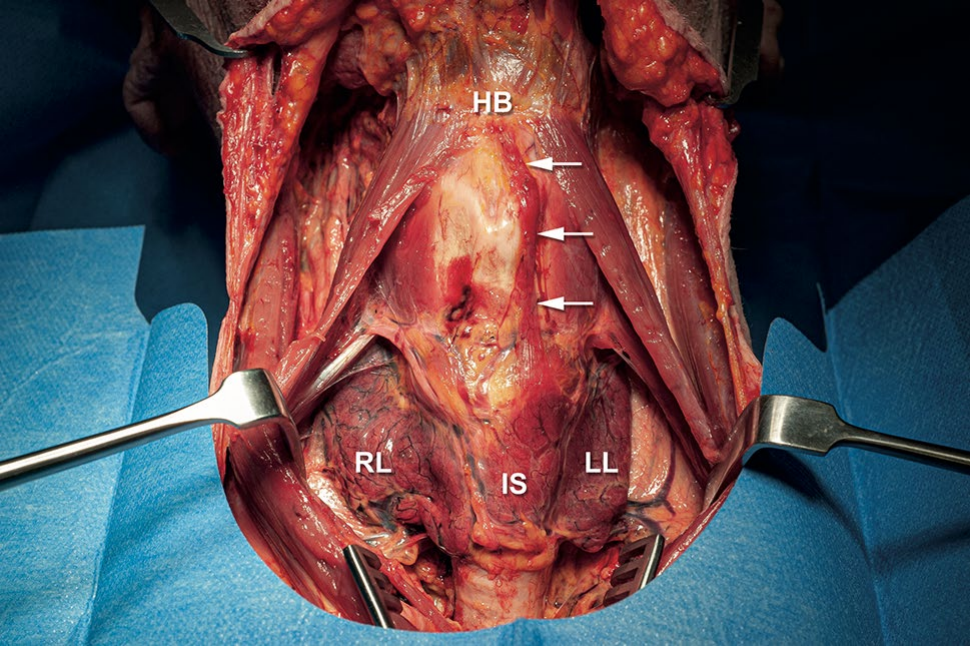
The same 80-year-old male cadaver after dissection. A left-sided PL (arrows) with connection to the hyoid bone that was previously detected with ultrasound was exposed. *RL* right lobe of the thyroid gland, *LL* left lobe of the thyroid gland, *IS* thyroid gland isthmus, *HB* hyoid bone

There was no difference in the location of the PL (*p* = 0.601, *χ*^2^ test, *n* = 34) or PL width (*p* = 0.425, Wilcoxon signed-rank test, two-tailed, *n* = 33) when assessed with ultrasound or during dissection. The distance to midline was significantly higher (*p* = 0.009, Wilcoxon signed-rank test, two-tailed, *n* = 33) when assessed with ultrasound compared to in situ. The comparison of ultrasound and anatomical PL characteristics is showcased in Table 6 (Figs. 1, 2).

**Table 6:** Comparison of parameters assessed with ultrasound and anatomical dissection

	Ultrasound (*n* = 34 true pos.)	Anatomy (*n* = 33 visualized)	
Side			
Left	16 (47.0%)	15 (45.5%)	*p* = 0.601
Median	4 (11.8%)	7 (21.2%)	
Right	14 (41.2%)	11 (33.3%)	
Width (mm)	6.92 ± 3.18	6.93 ± 3.11	*p* = 0.425
Distance to midline (mm)	5.31 ± 4.17	3.04 ± 3.21	*p* = 0.009

Data are presented in Values (M ± SD), frequencies and proportions (%)

## Discussion

### Prevalence of the pyramidal lobe

The PL is known as an ancillary lobe of the thyroid gland, correlating with the caudal end of the embryologic thyroglossal duct. Its prevalence in literature ranges from 35.7 to 60.0% in cadaver studies and from 12.3 to 61.0% when assessed during thyroid surgery [1–10]. In the present study, we observed a prevalence of 80%, which is markedly higher than what was previously reported. We believe that the methods applied in this study contributed to this finding and raise the possibility that PLs might have been overlooked beforehand. A crucial weakness in the design of previous studies regarding the PL prevalence was the definition of negative controls. Whereas other investigators used CT, anatomical dissection, or intraoperative visual identification, we histopathologically analyzed complete tissue blocks of the anterior neck region.

In contrast to the previous cadaveric studies conducted by Braun et al. [1], Ranade et al. [2], Ozgur et al. [3], Prakash et al. [4] and Milojevic et al. [5], we used fresh, non-embalmed cadavers. As fixation with formalin leads to decolorization and alters the tissue properties, the usage of fresh, non-embalmed cadavers enables a better differentiation of thyroid tissue from its surroundings. Additionally, the presence and location of the PL was indicated by ultrasound conducted prior to dissection. The microscopic examination of the tissue blocks in all cases in which the PL was not observed using ultrasound or on dissection further minimized the possibility of overlooking the PL. In the present study, one PL was not found during dissection and would have gone unnoticed without microscopic examination.

In comparison to Geraci et al., Zivic et al., Kim et al., Ryu et al. and Irawati et al., who reported the PL’s prevalence based on findings during thyroid surgery, we had the advantage of not being confined to a regular surgical area. To improve the exposure of the anterior cervical structures, the post-mortem nature of our study allowed us to perform larger skin incisions than normally required for hemi-/thyroidectomy in vivo. Furthermore, the patients included in these studies underwent thyroid surgery for various pathologies of the thyroid gland, potentially creating a selection bias [6–10]. While we cannot ensure the exact representation of the general Austrian population with our study sample, we aimed to minimize any sort of bias by randomly selecting body donors at our institution.

All individuals examined in this study were born before the compulsory iodization of salt (10 mg KI/kg salt) by law in Austria in 1963, which used to be an area of iodine deficiency and endemic goiter. Due to persistent deficiency the initial supplement was increased to 20 mg KI/kg in 1990 [28]. Considering the mean age of our study population at 81.3 ± 10.2 years, these body donors had already reached adult age by this time. As reported by Heinisch et al., goiter prevalence was expected to remain relatively unchanged in the population that had suffered from iodine deficiency prior to supplementation [30]. However, neither thyroid volume, nor thyroid parenchyma echogenicity, nor age interfered with the accuracy of PL detection according to regression analysis.

Despite the smaller sample size (*n* = 50) yielding lower statistical power in comparison to the aforementioned studies [1, 2, 4–10] (except Ozgur et al. [3], Table 1), the 95% confidence interval [68.9%; 91.1%] suggests a higher prevalence than formerly observed and strongly supports the opinion that the PL has to be considered a regular part of the thyroid gland [1, 10].

### Sonographic detection of the pyramidal lobe

It was previously shown that ultrasound offers good diagnostic value in the detection of the PL [8, 9]. However, these prior studies were conducted with an in-hospital population presenting with thyroid or neck pathology (e.g., known malignancy, neck mass, lymphadenopathy), potentially generating selection bias or afflicting anatomy. Additionally, surgical findings or the prevalence of the PL on CT was used as reference [8, 9, 23, 24]. To our knowledge, this is the first study investigating the sonographic detection of the PL in a post-mortem setting, confirmed by histopathological confirmation. With the methods applied, we were able to reliably exclude false-negative results.

Similarly to Kim et al. [24], who compared the detection rates of the PL of multiple investigators using ultrasound and CT, we investigated the impact of the examiner’s experience level. Examiner I is a radiologist specialized in small parts ultrasound with more than 15 years of experience. In contrast, examiner II had acquired basic head and neck ultrasound skills during medical school. Diagnostic performance for examiner I was sensitivity of 85.0%, specificity of 50.0%, PPV of 87.2%, NPV of 45.5% and accuracy of 78.0%. At a very basic level of training, examiner II achieved sensitivity of 42.5%, specificity of 60.0%, PPV of 81.0%, NPV of 20.7% and accuracy of 46.0%, not yielding a better discrimination than by chance. These widely differing results highlight the importance of a proficient examiner. Expert assessment, optimal adjustment of settings and adept handling of the ultrasound probe enable the differentiation of delicate structures such as the PL.

Examiner I`s diagnostic performance was comparable to preceding studies in terms of sensitivity (72.6–85.2%), PPV (85.3–93.3%) and accuracy (80.3–87.5%), (Table 7) [8, 9, 23, 24]. Regarding specificity (79.2–95.3%) and NPP (73.7–78.6%), however, we achieved considerably lower values at 50.0% and 45.5%, respectively [8, 9, 23, 24]. We believe the superior resolution of this ultrasound system to be in part responsible for the relatively high false-positive rate of 50% (5/10). As noted by Ryu et al. and Kim et al., longitudinally arranged muscular fibers or fibro-fatty tissue can mimic the presence of a PL on ultrasound and cause false-positive results [9, 24]. High-resolution imaging by modern ultrasound systems may enhance the discrimination of such longitudinally arranged structures and cause a false-positive result. Therefore, close attention should be paid to the echogenicity of the tissue in question.

**Table 7:** Comparison of diagnostic indices with previous studies

	Present study* (*n* = 50)	Kim et al. [22] (*n* = 160)	Kim et al. [8] (*n* = 132)	Ryu et al. [9] (*n* = 135)	Kim et al. [23] (*n* = 582)
Prevalence (%)	80.0	59.3	59.8	60.0	47.6
Sensitivity (%)	85.0	81.0	82.3	85.2	72.6
Specificity (%)	50.0	79.2	95.3	81.5	91.5
PPV (%)	87.2	85.3	93.3	87.3	89.3
NPV (%)	45.5	73.7	78.2	78.6	77.3
Accuracy (%)	78.0	80.3	87.5	83.7	82.1

*PPV*: positive predictive value, *NPV*: negative predictive value

*Investigation performed by examiner I

Another potential reason for the discrepancy in specificity is that Kim et al. and Kim et al. used the prevalence of the PL reported by CT as gold standard, but not surgical or anatomical confirmation [23, 24]. Although CT was previously shown to have favorable diagnostic value (sensitivity 91.4% and specificity 94.4%) when compared to surgical prevalence as gold standard, an uncertain number of false-positive and false-negative cases remain [9].

Despite the poor specificity observed in the present study, we advocate the screening and assessment of the PL in routine thyroid ultrasound as it can be detected with satisfactory sensitivity and provides additional information to the treating physician.

For clinical implication, a PL located along the former thyroglossal duct with separation from the main thyroid gland is of special interest. This was reported by Braun et al. in 1.7%, by Kim et al. in 3.8% and Ryu et al. in 5.2%, respectively [1, 8, 9]. Accounting for 4% (2/50) in the present study, these remnants of thyroid tissue pose a high risk of not being detected during surgery as they lack communication to the thyroid isthmus. In the two cases presented in this study, the separated PL was located ventrally on the thyrohyoid membrane and would have most likely been overlooked by a surgeon screening the isthmus for a protruding PL. Both cases were correctly identified by examiner I using ultrasound. In the case of a separated PL, imaging prior to surgery could yield decisive information.

### Limitations

Our study has certain limitations. This is a single-center study with a limited number of participants (*n* = 50) as restricted by the availability of body donors. Only two investigators with a substantially differing experience level performed ultrasound examination. Studies with a larger sample size and involving multiple investigators of similar experience could yield more generalizable data on the sonographic detection of the PL.

## Conclusion

The pyramidal lobe has a higher prevalence than previously considered and may consequently be regarded as a regular part of the thyroid gland. Physicians who are proficient in head and neck ultrasound can detect the PL with sufficient sensitivity and accuracy with high-resolution ultrasound. We therefore promote the assessment of the PL in routine thyroid ultrasound.

## Data Availability

The datasets generated and analyzed during the current study are available from the corresponding author on reasonable request.
